# Decreased Hyocholic Acid and Lysophosphatidylcholine Induce Elevated Blood Glucose in a Transgenic Porcine Model of Metabolic Disease

**DOI:** 10.3390/metabo12121164

**Published:** 2022-11-23

**Authors:** Jianping Xu, Kaiyi Zhang, Bintao Qiu, Jieying Liu, Xiaoyu Liu, Shulin Yang, Xinhua Xiao

**Affiliations:** 1 The Ministry of Health Key Laboratory of Endocrinology, Department of Endocrinology, Peking Union Medical College Hospital, Peking Union Medical College, Chinese Academy of Medical Sciences, Beijing 100730, China; 2 State Key Laboratory of Animal Nutrition, Ministry of Agriculture Key Laboratory of Animal Genetics Breeding and Reproduction, Institute of Animal Science, Chinese Academy of Agricultural Sciences, Beijing 100193, China; 3 Faculty of Gembloux Agro-Bio Tech, University of Liège, B5030 Gembloux, Belgium; 4 The Ministry of Health Key Laboratory of Endocrinology, Department of Central Laboratory, Peking Union Medical College Hospital, Peking Union Medical College, Chinese Academy of Medical Sciences, Beijing 100730, China

**Keywords:** high-fat high-sucrose diet, metabolic diseases, pig model, metabolome, lipidome

## Abstract

(1) Background: This work aims to investigate the metabolomic changes in PIGinH11 pigs and investigate differential compounds as potential therapeutic targets for metabolic diseases. (2) Methods: PIGinH11 pigs were established with a CRISPR/Cas9 system. PNPLA3^I148M^, hIAPP, and GIPR^dn^ were knocked in the H11 locus of the pig genome. The differential metabolites between and within groups were compared at baseline and two months after high-fat-high-sucrose diet induction. (3) Results: 72.02% of the 815 detected metabolites were affected by the transgenic effect. Significantly increased metabolites included isoleucine, tyrosine, methionine, oxoglutaric acid, acylcarnitine, glucose, sphinganines, ceramides, and phosphatidylserines, while fatty acids and conjugates, phosphatidylcholines, phosphatidylethanolamines, and sphingomyelins were decreased. Lower expression of GPAT3 and higher expression of PNPLA3^I148M^ decreased the synthesis of diacylglycerol and phosphatidylcholines. Accumulated ceramides that block Akt signaling and decrease hyocholic acid and lysophosphatidylcholines might be the main reason for increased blood glucose in PIGinH11 pigs, which was consistent with metabolomic changes in patients. (4) Conclusions: Through serum metabolomics and lipidomics studies, significant changes in obesity and diabetes-related biomarkers were detected in PIGinH11 pigs. Excessive fatty acids β-oxidation interfered with glucose and amino acids catabolism and reduced phosphatidylcholines. Decreased hyocholic acid, lysophosphatidylcholine, and increased ceramides exacerbated insulin resistance and elevated blood glucose. Phosphatidylserines were also increased, which might promote chronic inflammation by activating macrophages.

## 1. Introduction

With socio-economic progress, the incidence of chronic metabolic diseases is increasing. Diseases such as diabetes, obesity, and hypertension are major health problems for society. In recent years, omics technology (metabolomics, lipidomics, genomics, transcriptomics, proteomics, etc.) has shown rapid development as the preferred method for early disease diagnosis. Based on highly developed technologies related to liquid chromatography coupled mass spectrometry (LC-MS/MS), some targeted and non-targeted studies have identified specific biomarkers of obesity and diabetes [1]. The analysis of a large number of data sets generated by metabolomics and lipidomics provides a new clue for the role of metabolites in regulating insulin sensitivity [2].

The choice of experimental animal models for medical research directly affects the effectiveness of the tests. Most current studies selected mammal models such as rodents, which are not as close to humans as pigs in terms of genetic similarity and the size of organs. However, pigs obtained strong tolerance to a nutrient-rich diet in the process of domestication. Our previous study showed that although the body weight of pigs fed an over-nutrient diet for 23 months increased to more than three times that of normal pigs, they just present the symptoms of pre-diabetes through islet compensatory proliferation and expansion of subcutaneous fat [3]. Pig models constructed by genetic modification may be able to compensate for these weaknesses in rodent and domestic pig models.

Until now, three successful diabetic pig models with single gene overexpression have been reported. The first was the type-3 form of maturity-onset diabetes of the young (MODY3) model produced by overexpression of the dominant-negative mutant hepatocyte nuclear factor 1α with the CMV-I.E promoter [4]. The second was the islet β-cell dysfunction model which incretin function was impaired by overexpression of the dominant-negative glucose-dependent insulinotropic polypeptide receptor (GIPR^dn^) with the rat insulin 2 gene promoter [5]. The third was the permanent neonatal diabetes mellitus model overexpressing the mutant porcine insulin gene (INS^C94Y^) with rat insulin-2 gene promoter [6]. The following reports showed that the GIPR^dn^ pig exhibited the pathological features of pre-diabetes with reduced proliferation of pancreatic β-cells, impaired glucose tolerance, and increased branch-chain amino acids (BCAAs), which are biomarkers of insulin resistance. However, there was no β-cell apoptosis or fasted hyperglycemia, which led to macrovascular and microvascular complications accompanied by lipid toxicity and metabolic inflammation. 

To obtain an ideal pig model for metabolic disorders, we constructed multi-transgenic pig models considering multiple pathogenic factors. The susceptible genes related to glucose and lipid metabolism were considered. The liver plays an important role in glucose homeostasis through glycogen synthesis and storage during feeding and glycogenolysis and gluconeogenesis during fasting. The genetic variant of patatin-like phospholipase domain-containing 3 rs738409 C > G p.I148M (PNPLA3^I148M^), which leads to the substitution of methionine for isoleucine at the 148th residue, is widely associated with nonalcoholic fatty liver disease (NAFLD) in various races. In diabetic patients, abnormal overexpression of human islet amyloid polypeptide (hIAPP) led to the accumulation of toxic IAPP oligomers and β-cell apoptosis with age. In our previous study, PNPLA3^I148M^, hIAPP, and GIPR^dn^ were knocked-in at the H11 locos of the pig genome to achieve the PIGinH11 triple-transgenic pigs, in which PNPLA3^I148M^ was expressed by the promoter of porcine ApoE gene, and later two genes were expressed by the promoter of porcine insulin gene [7].

The PIGinH11 pig under the high-fat high-sucrose diet (HFHSD) for two months showed significant characteristics of metabolic diseases, such as abnormal glucose tolerance and obesity. Pre-pathological studies suggest that this porcine model has significant hepatic inflammation infiltration, similar to the pathology of human NAFLD. The current study found that some metabolites are significantly altered in patients with chronic disease. For example, it has been shown that bile acids are significantly reduced in patients with obesity and type-2 diabetes mellitus (T2DM) compared with normal subjects, and thus it can be used as an early warning indicator of metabolic diseases. Based on the above findings, we further performed metabolomic and lipidomic analysis to assess the metabolic abnormalities before and after HFHSD induction in PIGinH11 pigs compared to non-transgenic pigs, and selected differential metabolites that might be valuable for clinical diagnosis and therapy.

## 2. Materials and Methods

### 2.1. Animals

PIGinH11 pigs were prepared by CRISPR/Cas9 mediated gene knock-in. The human GIPR^dn^ and hIAPP which promote β-cell apoptosis, and human PNPLA3^I148M^ which enhances hepatic lipid deposition and insulin resistance, were transferred into pigs, as described previously [5]. Ten male PIGinH11 pigs 9-months of age were selected as the experiment group and ten age-matched male wild-type pigs were selected as controls. Tested pigs were housed in single pens with proper temperature and humidity. Pigs were fed with HFHSD (sucrose 37%, lard 10%, cholesterol 2%, basal 51%) for two months. 

### 2.2. Plasma Sample Collection and Biochemical Tests

Body weights were measured and blood samples were collected at the baseline (9-month-age) and after HFHSD induction. After 16 h of fasting, venous blood was collected from the anterior vena cava and centrifuged at 4 °C, 1000 g for 10 min to isolate plasma. Samples for the glucagon-like peptide 1 (GLP-1) test were stored in a BDtm p800 blood collection tube at −80 °C in the refrigerator until the assay. Plasma biochemical parameters including blood glucose, insulin, and GLP-1 were tested using an automatic biochemical analyzer (AU480, Olympus Co., Tokyo, Japan).

### 2.3. Metabolite Extraction

Plasma samples (100 μL each) were precipitated by the addition of three volumes of acetonitrile (ACN) (polar ionic mode) or isopropyl alcohol (IPA) (lipid mode) precooled to −20 °C. Samples were vortex mixed for 1 min. After 10 min of incubation at room temperature, samples were stored overnight at −20 °C to improve protein precipitation and then centrifuged at 12,000× *g* for 20 min. The supernatant was collected and awaiting HPLC-MS analysis. Quality control (QC) sample was prepared by mixing an equal volume (50 µL) of each test sample. The QC sample was injected six times at the beginning of the run to ensure the equilibration of the UPLC/Q-TOF MS system and then between every 10 samples throughout the run to ensure the consistency of the analysis.

### 2.4. Untargeted HPLC-MS Analysis

The metabolomic analysis was performed on a Nexera X2 LC-30AD (Shimadzu, Kyoto, Japan) coupled with a TripleTOF 5600+ (SCIEX, Framingham, MA, USA) in both positive and negative ionization modes. For metabolomic profiling, the column was a 2.1 mm × 100 mm, 1.8 μm, HSS T3 column (Waters Ltd., Elstree, UK) and the temperature was maintained at 45 °C. The flow rate was 250 μL/min. Mobile phase A consists of 0.1% formic acid in H2O and mobile phase B 0.1% formic acid in ACN. The separation was conducted under the following gradient: 0 min 5% B; 0–1.5 min 20% B; 1.5–15 min 90% B; 15–18 min 100% B; 18–18.1 min 5% B; 18.1–20 min 5% B. 

For lipidomic profiling, the column was a 2.1 mm × 100 mm, 2.6 μm, C18 100Å column (Phenomenex, Torrance, CA, USA), and the temperature was maintained at 45 °C. Mobile phase A consists of H2O/ACN/MeOH (3:1:1, *v*:*v*:*v*) mixed with 5 mM ammonium acetate, and mobile phase B consists of IPA mixed with 5 mM ammonium acetate. The flow rate was 300 μL/min. The separation was conducted under the following gradient: 0 min 5% B; 0–1 min 5% B; 1–16 min 95% B; 16–18 min 95% B; 18–18.1 min 5% B; 18.1–22 min 5% B. 

For both metabolomic and lipidomic profiling, the Time-of-Flight (Q-TOF) Mass Spectrometry was operated in electrospray ionization by using IDA high-sensitivity scanning mode with the following parameters: curtain gas, 35 psi; ion source gas 1, 60 psi; ion source gas 2, 60 psi; temperature, 550 °C; ion spray voltage floating, 5.5 kV for the positive mode and the negative was 4.5 kV. The scanning time is 22 min; the scan range of the positive mode was from 100–1250 m/z for MS scan and 50–1250 m/z for MS/MS scan and the negative was 100–1200 m/z for MS scan and 50–1200 m/z for MS/MS scan; 12 ions from the MS scan were selected for the MS/MS scan. The MS/MS accumulation time is 0.05 s, the collision energy is 40, and the collision energy range is the theoretical frequency ±20.

### 2.5. Data Processing and Statistical Analysis

Raw data were imported to the Progenesis QI (waters) for peak alignment to obtain a peak list containing the retention time, m/z, and peak area of each sample. By using retention time and the m/z data pairs as the identifiers for each ion, we obtained ion intensities of each peak and generated a matrix containing arbitrarily assigned peak indices (retention time-m/z pairs), ion intensities (variables), and sample names (observations). The matrix was further reduced by removing peaks with missing values in more than 80% of samples and those with isotope ions from each group to obtain consistent variables. The CV (coefficient of variation) of metabolites in the QC samples was set at a threshold of 30%, as a standard in the assessment of repeatability in metabolomics data sets. Metabolites selected for further statistical analysis were identified based on variable importance in the projection (VIP) threshold of one of the tenfold cross-validated OPLS-DA models, which was validated at a univariate level with an adjusted *p* < 0.05. The HMDB database (http://www.hmdb.ca, accessed on 5 February 2022), KEGG database (http://www.genome.jp/kegg/, accessed on 5 February 2022), and NIST were used to align the molecular mass data (m/z) to identify metabolites (polar ionic mode). The HMDB database (http://www.hmdb.ca, accessed on 5 February 2022), Lipid maps Structure Database (LMSD), and NIST were used to align the molecular mass data (m/z) to identify metabolites (lipid mode). The mass error used was 0.005 Da for ms1 and 15 ppm for ms2. The well-established online analyzing platform MetaboAnalyst 5.0 published by Pang et al. (https://www.metaboanalyst.ca, accessed on 5 February 2022) was used for the Enrichment Analysis and heatmaps. Statistical analysis of the biomarkers and specific metabolites was performed using the Student’s *t*-test and *p*-values were adjusted with a false discovery rate (FDR). The adjusted *p*-values were represented by *adj. p*.

### 2.6. Immunoblotting

Tissue samples were collected after HFHSD induction and stored at −80 °C, while 100 mg of liver or skeletal muscle tissues were ground in liquid nitrogen and total proteins were extracted using the Tissue Protein Extraction Reagent (Thermo #78510). cOmplete Tablets (Roche 04693159001) and PhosSTOP EASYpack (Roche 04906837001) were added to prevent protein degradation and 30 μg of denatured total protein were loaded to each line. Antibodies recognizing PNPLA3 (Abcam), phosphor-Akt Ser473 (CST #9271), total Akt (CST #4685), GPAT3 (Bioss bs-6547R), MOGAT2 (Proteintech 19514-1-AP), and TMEM16F (Proteintech 20784-1-AP) were used to detect the relative protein expressions and GAPDH (CST#2118) were used as house-keeper. Anti-rabbit IgG, HRP-linked Antibody (CST #7074) was used as the second antibody. The grayscale density of chemiluminescence images was analyzed using ImageJ software. Statistical analysis was performed by Prism8 using the Student’s *t*-test.

## 3. Results

### 3.1. Weight Change and Plasma Biochemical Parameters

Comparisons of body weight and plasma biochemical parameters related to diabetes were made between wild-type and PIGinH11 pigs at baseline (WT-0 and TG-0) and after two months of HFHSD feeding (WT-2 and TG-2) (Table 1). The results showed a significant increase in body weight in both groups after two months of HFHSD feeding. There was no significant difference between the groups. 

There was a significant increase in blood glucose at baseline in PIGinH11 pigs compared to the wild-type pigs. Further HFHSD feeding for two months in both groups resulted in a significant increase in blood glucose in wild-type pigs, but fasting blood glucose in PIGinH11 pigs was not significantly changed, with mean values greater than 7.0 mmol/l. Insulin levels were significantly higher in the TG-0 compared to the WT-0, indicating significant insulin resistance in the PIGinH11 pigs. Insulin levels in the wild-type pigs increased significantly after HFHSD feeding, comparable to that of the PIGinH11 pigs. GLP-1 level was lower in TG-0 than that in WT-0 at baseline and was decreased in wild-type pigs after HFHSD feeding. No significant differences were found in GLP-1 levels between WT-2 and TG-2.

There was no significant difference between groups at baseline in terms of blood lipids. Total cholesterol and HDL cholesterol were both increased in PIGinH11 pigs and wild-type pigs after HFHSD feeding.

### 3.2. Metabolomic and Lipidomic Profiling of Pig Plasma

**Figure 1:**
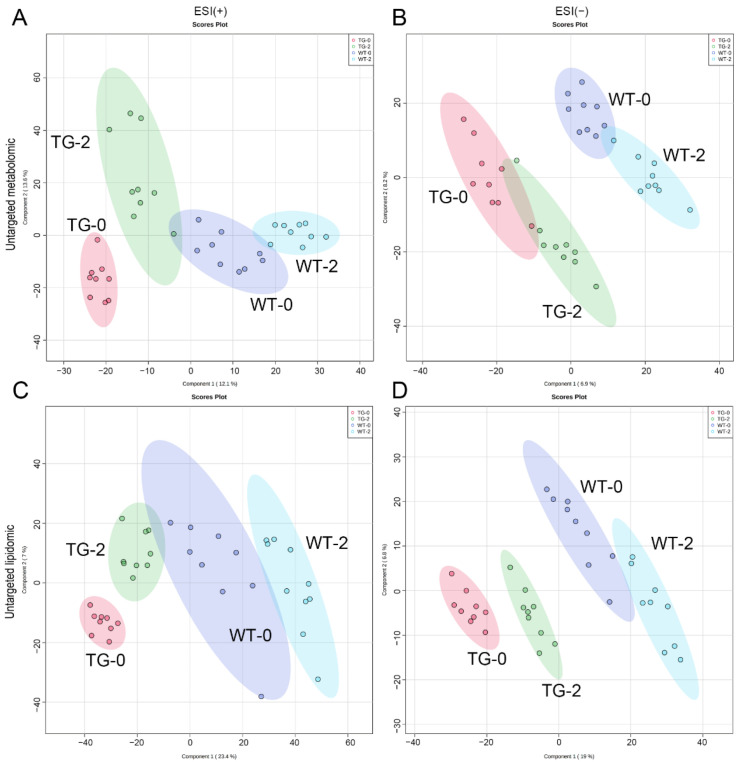
Principal component analysis (PCA) were performed by positive and negative ion modes of the untargeted metabolome (**A**,**B**) and lipidome (**C**,**D**). ESI (+), positive ion modes, ESI (−), negative ion modes.

To further clarify the metabolic changes in PIGinH11 pigs, we analyzed the metabolome and lipidome of wild-type pigs and PIGinH11 pigs using an LC-MS approach. Plasma samples were collected from wild-type and PIGinH11 pigs fed with a normal diet at baseline (WT-0 and TG-0), and from the same experimental group after being fed with HFHSD for 2 months (WT-2 and TG-2). There were 3009 and 4170 features detected in the positive and negative ion modes of the untargeted metabolome, while 3865 and 2063 features were detected in positive and negative ion modes of the untargeted lipidome. Principal component analysis (PCA) showed significant separation of the four groups in both positive and negative ion modes of the untargeted metabolomic and lipidomic profiles, indicating that there were significant metabolic differences between wild-type and PIGinH11 samples before or after the HFHSD induction (Figure 1).

Orthogonal partial least squares-discriminant analysis (OPLS-DA) score plots were applied to identify the differential metabolites or lipids among the different groups. Significant differential variables contributing to the separation of groups were selected based on the following criteria: VIP > 1 in OPLS-DA models and *p* < 0.05 (Tables S1–S4). There were 815 differential metabolites or lipids between at least one compared combination among WT-2/WT-0, TG-2/TG-0, TG-0/WT-0, and TG-2/WT-2. The statistical results of the differential metabolites were shown in Table 2.

**Figure 2:**
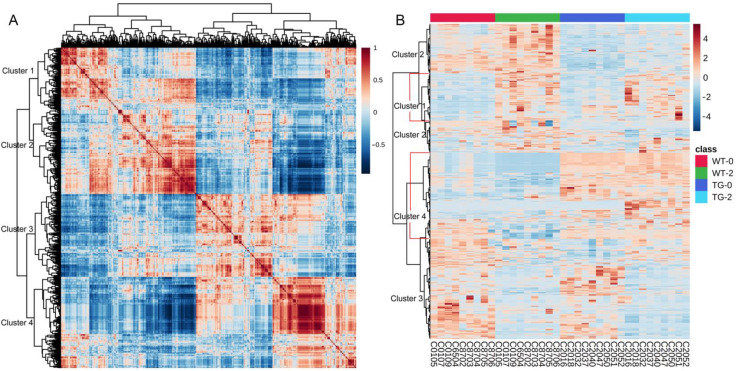
Hierarchical clustering of the 815 differential features comparing the four groups. (**A**) The correlation map between the 815 differential metabolites and lipids. (**B**) Heatmap showing the abundance of the 815 differential metabolites and lipids in each sample.

Hierarchical clustering divided the 815 differential features into four branches (Figure 2), which represent 131 up-regulated (Figure 2 cluster 1) and 211 down-regulated (Figure 2 cluster 3) features caused by HFHSD diet induction, and 242 up-regulated (Figure 2 cluster 4) and 231 down-regulated (Figure 2 cluster 2) features caused by the transgenic effect. Within the features affected by dietary effect, 44 features had a higher abundance in PIGinH11 pigs than in wild-type pigs (*p* < 0.05) in cluster 1, and 70 features had a lower abundance in PIGinH11 pigs than in wild-type pigs (*p* < 0.05) in cluster 3. In summary, there were 587 features affected by the transgenic effect, accounting for 72.02% of the total difference metabolites.

**Figure 3:**
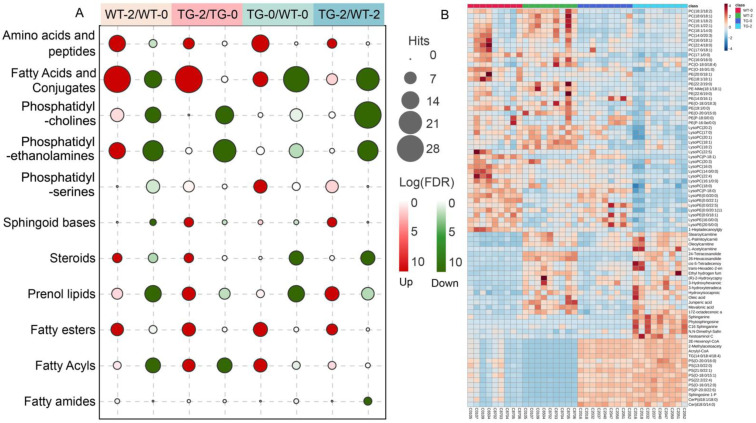
Enrichment analysis (**A**) and heatmap (**B**) exhibited the differential metabolites, and lipids in the four groups of pig plasma samples. PC, Glycerophosphocholines. PE, Glycerophosphoethanolamines. PS, Glycerophosphoserines.

In the 815 differential features, 178 items were not checked with matched records in the HMDB, PubChem, ChEBI, KEGG, or METLIN databases, 134 items were not included in the HMDB database, and 140 items could not be accurately determined. The remaining 363 items were uniquely identified in the HMDB database for enrichment analysis with online metabolomics data analysis platform (MetaboAnalyst 5.0). The metabolite set library, main-class 464 data sets of metabolic or lipid sets based on chemical structure, were selected for enriching the up-regulated and down-regulated features between WT-2 versus WT-0, TG-2 versus TG-0, TG-0 versus WT-0, and TG-2 versus WT-2 (Figure 3, Table S5). Amino acids and peptides were the only data sets for metabolites that significantly differed between groups (*p* < 0.05). TG-0/WT-0 and WT-2/WT-0 enriched the most abundant 13 and 12 metabolites, respectively (Figure 3A). L-leucine, L-isoleucine, kyotorphin, suberylglycine, and tryptophyl-histidine were mainly correlated with dietary effects and were up-regulated in both WT-2/WT-0 and TG-2/TG-0. The ratios of betaine, L-methionine, and L-tyrosine ranged from 1.42 to 1.87 in TG-0/WT-0 and TG-2/WT-2 while creatine, L-proline, L-arginine, and L-glutamine were also up-regulated in the TG-0/WT-0 group, which means the increases of these metabolites might due to the transgenic effect.

The enriched datasets of lipids mainly included fatty acids and conjugates, phosphatidylcholines (PCs) or lysophosphatidylcholines (LysoPCs), phosphatidylethanolamines (PEs) or lysophosphatidylethanolamines (LysoPEs), phosphatidylserines (PSs), sphinganine base, steroids, prenol lipids, fatty esters, fatty acyls and fatty amides (Figure 3A). Fatty acids and conjugated compounds, enriched the most abundant lipids, were mainly increased with dietary induction and down-regulated by transgenic effect. There were 27 and 28 lipids up-regulated in wild-type and PIGinH11 pigs by the effect of HFHSD, accompanied by 13 and 3 lipids down-regulated by HFHSD, respectively. In terms of the transgenic effect, 10 or 6 lipids showed higher abundance in PIGinH11 pigs than that in wild-type pigs, with or without dietary intervention. Interestingly, 25 and 20 lipids were significantly lower in PIGinH11 pigs before and after HFHSD, respectively. 

Plasma PCs and PEs were affected by both dietary effects and transgenic effects. 7 PCs and 9 PEs were significantly lower in TG-0 than in WT-0 and the number increased to 27 for PCs and 17 for PEs, comparing TG-2 and WT-2, which represented the transgenic effects. The dietary intervention led to 8 up- and 11 down-regulated PCs and 12 up- and 17 down-regulated PEs in wild-type pigs, respectively. The dietary effect on PIGinH11 pigs was also significant, determined by 14 decreased PCs and 20 decreased PEs. Furthermore, dietary induction and transgenic effects increased plasma fatty esters. There were 7, 8, 9, and 6 of these lipids up-regulated in the WT-2/WT-0, TG-2/TG-0, TG-0/WT-0, and TG-2/WT-2, respectively. Interestingly, HFHSD intervention increased some species of steroids, but transgenic reduced them. Five steroids were up-regulated in both WT-2/WT-0 and TG-2/TG-0 and nine steroids were down-regulated in both TG-0/WT-0 and TG-2/WT-2. Dietary intervention and transgenic together increased sphinganine bases, five of which up-regulated in the TG-2 group compared with both TG-0 and WT-2 groups, respectively.

According to the enrichment analysis, the transgenic effect had a great impact on lipid metabolism. Heatmap in Figure 3B showed the differential lipids between the four groups. Compared with wild-type pigs, the plasma level of PCs, PEs, LysoPCs, and LysoPEs were significantly lower in TG-0 at the baseline. HFHSD intervention significantly decreased the level of these lipids in both wild-type and PIGinH11 pigs. However, the most notable decrease in those glycerophospholipids was found in PIGinH11 pigs after HFHSD induction. Meanwhile, fatty esters, saturated fatty acids, and sphingomyelins of TG-0 samples were significantly higher than that of WT-0 at baseline. These lipids were also increased due to HFHSD intervention in wild-type and PIGinH11 pigs. Therefore, TG-2 samples had significantly higher levels of these lipids than both WT-2 and TG-0 samples. Acyl-CoA, PSs, sphingosine 1P, and ceramides of the TG-0 group were significantly higher than that of the WT-0 group at baseline. This group of lipids was decreased in wild-type pigs after HFHSD but was increased in PIGinH11 pigs after HFHSD. Correspondingly, the ratios of TG-2/WT-2 for these lipids were further increased. The abundance of ceramide (Cer(d18:0/14:0)) and ceramide phosphate (CerP(d18:1/18:0)) in PIGinH11 samples were 83 to 184 folded than those in the wild-type samples before or after dietary induction, leading to a strong inhibitory effect on insulin signaling according to previous studies. Acylcarnitines, the intermediate metabolites of transporting fatty acids into the mitochondria for β-oxidation, were significantly higher in TG-0 than in WT-0. HFHSD induction significantly increased acylcarnitines in WT-2 samples as well, indicating an energy substrate preference shift toward fatty acids and accompanied insulin resistance due to both transgenic and dietary effects.

**Figure 4:**
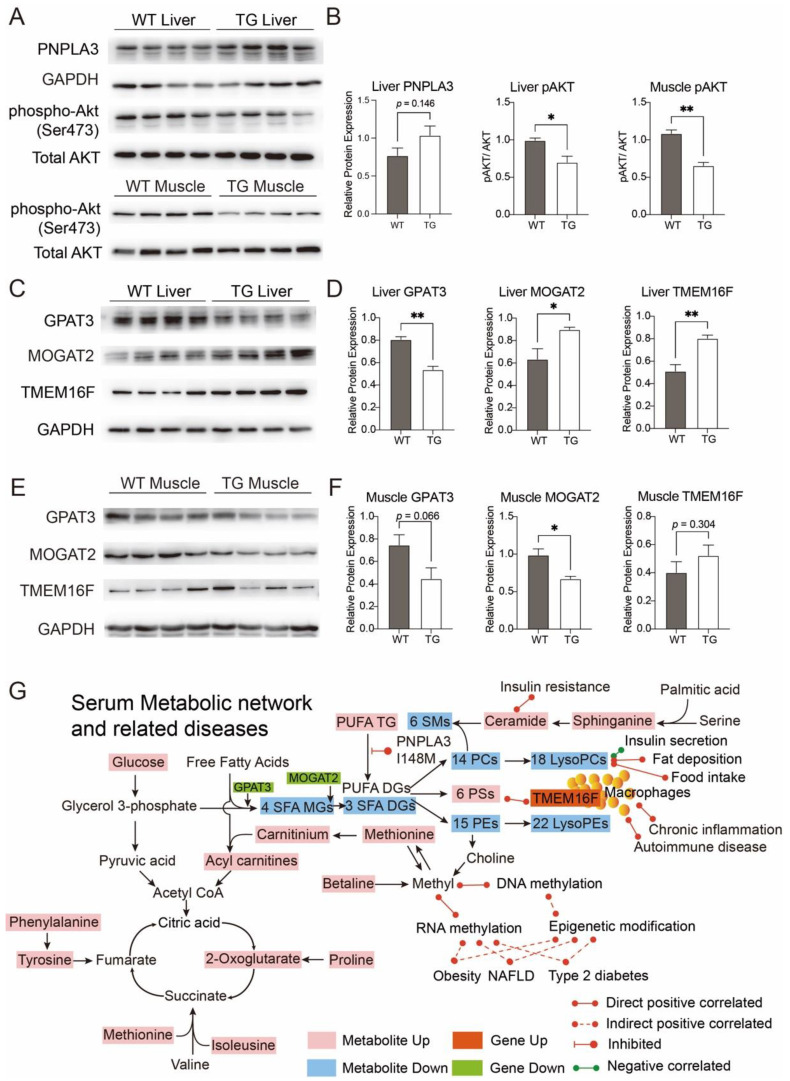
Immunoblotting results and the metabolic network of differential metabolites between PIGinH11 (TG) and wild-type (WT) pigs after 2-months of HFHSD. (**A**,**B**) Protein expression of total PNPLA3 (porcine PNPLA3 and humanized PNPLA3^I148M^) was detected using liver samples. The degree of Akt activation was investigated by detecting phosphor-Akt. The lower ratio of phosphorylated Akt Ser473 to total Akt in TG samples indicated suppressed Akt signaling. The protein expressions of GPAT3, MOGAT2, and TMEM16F were detected using liver (**C**,**D**) and skeletal muscles (**E**,**F**) from TG and WT pigs. The differential metabolites’ network was shown in (**G**). GPAT3, Glycerol-3-Phosphate Acyltransferase 3, also refers as MAG1. MOGAT2, Monoacylglycerol O-Acyltransferase 2. TMEM16F, anoctamin 6, also refers as ANO6. Data were shown as mean ± SD, n = 4 per group, * *p* < 0.05, ** *p* < 0.01.

Based on the above results, we detected the phosphorylation state of serine/threonine kinase Akt, which is the crucial regulator in insulin signaling. Indeed, the ratio of phospho-Akt at Ser473 to total Akt was significantly lower in the liver and skeletal muscle samples from PIGinH11 pigs, indicating their suppressed Akt activation and impaired insulin signaling (Figure 4A,B). To determine the potential source of these differential lipids, we detected the protein expression of the relevant enzymes in the liver and skeletal muscle samples. Protein expression of total PNPLA3, including the endogenous porcine PNPLA3 and the humanized PNPLA^3I148M^ that we knocked in pig’s liver, was slightly higher in PIGinH11 pigs (*p* = 0.146). Protein expression of glycerol-3-phosphate acyltransferase 3 (GPAT3), which catalyzes diacylglycerol synthesis, was significantly lower in the liver of the PIGinH11 pigs (*p* < 0.01) and slightly lower in skeletal muscle (*p* = 0.066), compared with wild-type samples. While the expression of monoacylglycerol o-acyltransferase 2 (MOGAT2), which catalyzes triglyceride synthesis, was significantly down-regulated in PIGinH11 pigs’ skeletal muscle (*p* < 0.05), but up-regulated in their liver (*p* < 0.05) (Figure 4C–F). Notably, the expression of anoctamin 6 (ANO6, also refers to TMEM16F) which promotes the translocation of PSs to the outer plasma membrane was significantly higher at the protein level in the liver and muscle samples from PIGinH11 pigs (Figure 4C–F). Based on the differential metabolites and lipids, we constructed a pattern of metabolic changes in PIGinH11 pigs, which was high relative to patients with metabolic diseases (Figure 4G).

### 3.3. Biomarkers of Obesity and Diabetes in PIGinH11 Pigs

**Figure 5:**
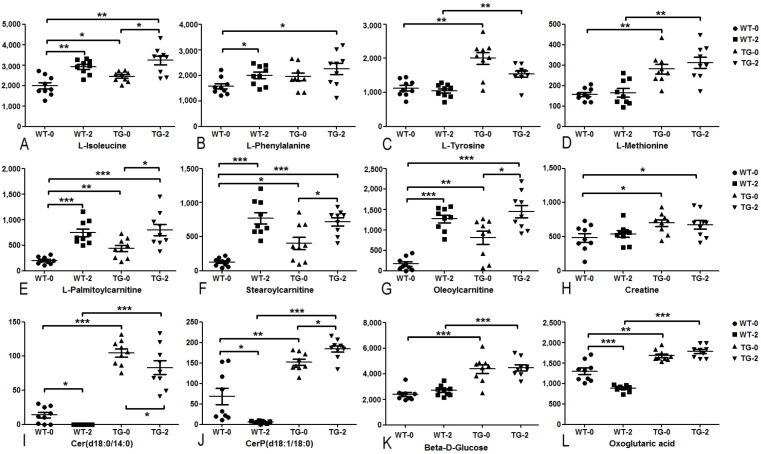
The abundance of commonly accepted biomarkers for diabetes and/or obesity-related disease in the four groups of pig plasma samples. (**A**–**D**,**H**) for amino acids, (**E**–**G**) for acylcarnitines, (**I**,**J**) for ceramides, (**K**) for glucose, (**L**) for oxoglutaric acid. * *adj. p* < 0.05, ** *adj. p* < 0.01, *** *adj. p* < 0.001.

Several metabolites widely accepted as biomarkers for diabetes or obesity-related diseases were detected as well in PIGinH11 pig models in this study. Figure 5 showed the abundance of these biomarkers in the four groups of pig plasma samples. Among amino acids, L-isoleucine, L-phenylalanine, L-tyrosine, L-methionine, and creatine of PIGinH11 pigs (Figure 5A–D,H) were significantly higher than the corresponding levels of wild-type pigs, with dietary induction significantly increasing the former two in the wild-type groups. Acylcarnitines were affected by both transgenic and dietary effects. Before the dietary intervention, the level of L-palmitoylcarnitine, stearoylcarnitine, and oleoylcarnitine in the TG-0 group were significantly higher than those in the WT-0 group (Figure 5E–G). The dietary intervention significantly increased the levels of the three acylcarnitines in the WT groups but remain unchanged in PIGinH11 pigs. Ceramide (d18:0/14:0) and ceramide phosphate (d18:1/18:0) were affected by both transgenic and dietary effects (Figure 5I,J), both of which were significantly higher in PIGinH11 pigs before and after HFHSD, while dietary intervention significantly down-regulated ceramide (d18:0/14:0) in both wild-type and PIGinH11 pigs. Ceramide phosphate (d18:1/18:0) decreased significantly comparing WT-2 with WT-0 but was increased comparing TG-2 with TG-0. Interestingly, ceramide (d18:0/14:0) and ceramide phosphate (d18:1/18:0) were almost undetectable in the WT-2 group. Notably, glucose and oxoglutaric acid were mainly affected by the transgenic effect (Figure 5K,L). The levels of glucose and oxoglutaric acid in PIGinH11 pigs were significantly higher than those in the wild-type pigs before and after HFHSD. Dietary induction significantly reduced oxoglutaric acid in wild-type pigs.

**Figure 6:**
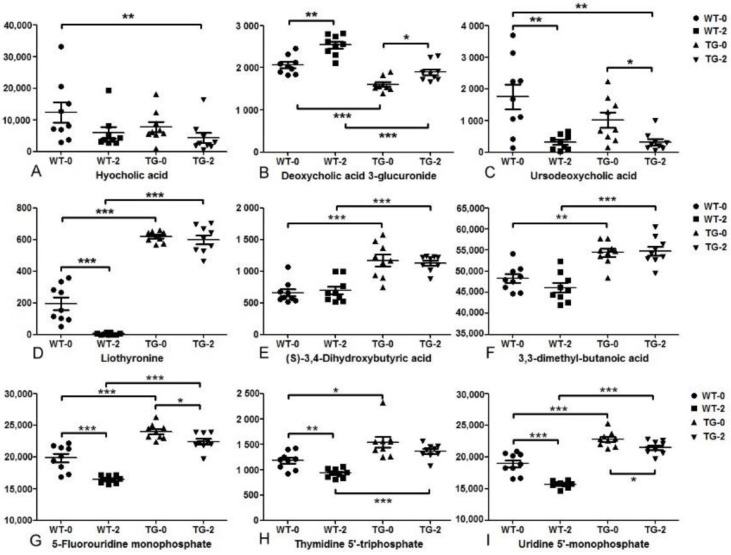
The metabolites and lipids that significantly altered by transgenic effect. (**A**–**C**) for bile acids, (**D**) for liothyronine, (**E**,**F**) for butyric acid derivatives, (**G**–**I**) for nucleotides. * *adj. p* < 0.05, ** *adj. p* < 0.01, *** *adj. p* < 0.001.

To provide detailed identification of the characteristics of PIGinH11 pigs’ metabolome and lipidome in the progression of metabolic disease, the features significantly altered in PIGinH11 pigs but not enriched in the datasets were considered as well (Figure 6, Table S6). Three of the classic bile acids, including hyocholic acid, deoxycholic acid 3-glucuronide, and ursodeoxycholic acid, were simultaneously affected by both transgene and dietary induction (Figure 6A–C). Hyocholic acid, the most abundant bile acid in pigs, was slightly lower in WT-2 and TG-0 compared with WT-0 (not significant). However, dietary induction significantly decreased hyocholic acid in TG-2 compared with WT-0 (*adj. p* < 0.01). Another two bile acids, deoxycholic acid 3-glucuronide, and ursodeoxycholic acid were significantly decreased by the transgenic effect, with dietary induction increasing the former and decreasing the latter. Liothyronine, which regulates energy metabolism, was significantly increased in PIGinH11 pigs and kept at a similar level after dietary induction, but decreased almost undetectably after HFHSD in the wild-type pigs (Figure 6D). The 3, 4-dihydroxybutyric acid and 3,3-dimethylbutyric acid that mainly originated from intestinal microbial fermentation had little changes due to dietary induction, but these two short-chain fatty acid derivatives were significantly increased by transgenic effect (Figure 6E,F). Three nucleotides including 5-fluorouridine monophosphate, thymidine 5’-triphosphate, and uridine 5’-monophosphate had different change modes by transgene and dietary induction (Figure 6G–I). The transgenic effect significantly increased these three nucleotides, while short-term dietary induction decreased them.

## 4. Discussion

T2DM is a complex disease caused by multiple factors, including genetic variations, unhealthy diet, and lack of exercise, leading to disorders of glucose and lipid metabolism in multiple tissues. The circulatory system is the main executor of the body’s material transport function, and can also transmit regulatory signals between tissues. Therefore, studying plasma metabolomics and lipidomics is an important way to understand metabolic homeostasis and inter-organizational communication disturbances during the development of diabetes. PIGinH11 pigs showed hyperglycemia before and after dietary intervention and 587 amino acids, fatty acids, phospholipids, bile acids, and hormones were altered in their plasma, which were related to transgenic effects, and these changes were highly similar to those in T2DM or NAFLD patients. 

Firstly, the biomarkers associated with obesity, NAFLD and T2DM, were exhibiting the same tendency as patients in PIGinH11 pigs. The accumulation of BCAAs [8], aromatic amino acids (AAAs), acylcarnitines, and tricarboxylic acid cycle intermediates have been used as clinical biomarkers of insulin resistance [2]. In the 1960s, elevated BCAAs have been found to be closely related to insulin resistance, and can be used to predict insulin resistance and diabetes even 20 years before clinical manifestations, which were more than 10 years earlier than other biomarkers. Sun et al. [9] conducted a systematic study on 982 studies related to metabolic predictors of diabetes and conducted a meta-analysis of data from 46 studies after screening. Their results showed that elevated BCAAs and AAAs may increase the risk of T2DM. The combination of increased isoleucine, phenylalanine, and tyrosine led to a 43% increased risk of T2DM. Lipid metabolite analysis showed that high acetylcarnitine C2 was predictive of impaired glucose tolerance and T2DM [10]. Elevated palmitic, lauric, stearic, linoleic acids, and depleted short-chain fatty acids in plasma were characterized in T2DM patients [11]. In this study, the BCAAs (isoleucine), AAAs (phenylalanine, tyrosine, and methionine), fatty acids (oleic acid, stearic acid), acylcarnitines ((S)-carnitinium, L-acetylcarnitine, L-palmitoylcarnitine, stearoylcarnitine, and oleoylcarnitine), ceramide (ceramide (d18:0/14:0), ceramide phosphate (d18:1/18:0)), and α-ketoglutarate and glucose in PIGinH11 pigs were significantly higher than those of WT pigs, which were consistent with the insulin resistance and diabetes of patients. As signal molecules, BCAAs regulate glucose and lipid metabolism through specific signal networks, especially the PI3K-AKT-mTOR signal pathway [12]. A mouse study showed that BCAA inhibited Akt2 activation through mTORC1 and mTORC2 signals for a long time, resulting in liver metabolic disorder and severe liver insulin resistance [13]. Therefore, the changes in serum BBCAs in PIGinH11 pigs may be related to the inhibition of the AKT signal pathway in the liver and skeletal muscle. 

Methionine and betaine were the main methyl donors and the former is crucial for carnitine synthesis, which promotes mitochondrial fatty acids transport. Both transgenic and dietary effects increased plasma methionine. The level of methionine level just slightly increased 1.11 and 1.05 times (*p* > 0.05) in WT and PIGinH11 pigs, responding to HFHSD. However, the transgenic effect significantly increased betaine, methionine, carnitine, and acetylcarnitine in pigs. For instance, the plasma abundance of methionine, (S)-carnitinium, and L-acetylcarnitine of PIGinH11 pigs were 1.78, 1.25, and 2.19 times of the WT group at the baseline, while they increased to 1.89, 2.30, and 5.53 times after HFHSD, respectively. These results indicated that there might be a step-by-step accumulation in the production of methionine, carnitine, and L-acetylcarnitine. Fatty acids and glucose are in a competitive relationship in oxidative energy supply [14]. Excessive fatty acid oxidation might be one of the important reasons that lead to glucose homeostasis disturbance and insulin resistance in PIGinH11 pigs. On the other hand, the increase of methyl donors can regulate gene expression through epigenetic modifications such as DNA and RNA methylation, and promote the occurrence and development of diseases such as obesity, NAFLD, and T2DM. Previous studies have shown that restricting the intake of methionine can improve metabolic disorders [15].

Secondly, phospholipid metabolism was disordered in PIGinH11 pigs, resulting in impaired cellular function and inflammation [16]. Phospholipids, including glycerophospholipids and sphingomyelins (SMs), are important components of the cellular membrane and important molecules in the regulation of signal transduction, cell proliferation, apoptosis, and inflammation. The most abundant phospholipid in mammalian membrane structures is PCs (40–50%), followed by PEs (17–25%) which can be enriched up to 35–40% in the mitochondria. SMs varied greatly between organelles, ranging from only 2% in the inner and outer mitochondrial membranes to 16–23% in lysosomes and plasma membranes. The distribution of phospholipids in the organelle bilayer membrane has an asymmetric pattern, with PCs and SMs enriched in the bilayer outer leaflet, while PEs and PSs enriched in the inner layer. 

Epidemiological studies showed that PCs, PEs, and sphingolipids are generally down-regulated in the development of obesity, NAFLD, and T2DM [17,18,19,20,21]. In PIGinH11 pigs, 22 species of fatty acids and conjugates, 14 PCs, 18 lysoPCs, 15 PEs, 22 lysoPEs, and 6 sphingomyelins were down-regulated. Among them, the SFAs and MUFAs containing 16C, 18C, or 20C, and the PUFAs of 18C, 20C, or 22C constituted the main parts. These results were in line with the studies on the target genes or mutations in humans and other models. Among the 6 PCs and 4 SMs detected in GIPRdn pigs, only 2 PCs and 1 SM were lower than those of wild-type controls at 2.5 months of age. However, all these phospholipids were lower in GIPRdn than those in controls at 5 months of age, showing a trend of progressive down-regulation with insulin resistance [22]. The PNPLA3^I148M^ mutant plays an important role in the development of NAFLD by remodeling the hepatic lipidome. Studies have shown that the noninvasive ClinLipMet score which incorporated the PNPLA3^I148M^ mutation can identify non-alcoholic steatohepatitis (NASH) patients more accurately than other NASH score systems or MS-based analyses. With the progression of liver diseases (from healthy to NAFLD and NASH), plasma triglycerides (TGs) were continuously up-regulated, while phospholipids and sphingolipids including PCs, LysoPCs, Pes, lysoPEs, and SMs, were gradually down-regulated [23]. Introduction of mutant I148M of PNPLA3 in human hepatocytes reduces the hydrolysis of intracellular polyunsaturated fatty acyl TGs and reduces the content of diacylglycerols (DGs) thereby reducing PC synthesis using it as a substrate, while the saturated fatty acids palmitic acid and oleic acid increase [24]. This result was consistent with our finding that plasma PCs were significantly down-regulated, while oleic and stearic acids were up-regulated in PIGinH11 pigs. GPAT3 protein expression was significantly reduced in both the liver and skeletal muscles of PIGinH11 pigs, leading to decreased DGs synthesis, while MOGAT2 which catalyzes TGs synthesis was increased in the liver but decreased in the muscles of PIGinH11 pigs. Therefore, we speculated that the decreased plasma monoacylglycerols (MGs) and DGs in PIGinH11 pigs might be caused by the oxidation of palmitic, stearic, and oleic acids that are associated with insulin resistance. In addition, PNPLA3^I148M^ inhibited the decomposition of polyunsaturated TGs containing 18C, 20C, and 22C and reduced the generation of DGs required for the synthesis of PCs and PEs. The decrease of PC in plasma limited the synthesis of sphingomyelin from ceramides, resulting in the accumulation of ceramides and sphinganines. Ceramide mediates the inhibition of AKT signal pathway through protein phosphatase 2A [25] and protein kinase Cζ [26], leading to insulin resistance. 

Previous research has shown that plasma LysoPCs levels are significantly reduced in obesity and T2DM patients [27]. Lysophospholipids are required to maintain homeostasis of many physiological processes such as nervous system function, vascular development, and reproduction [28]. Reports show that LysoPCs induce insulin secretion from pancreatic cells. LysoPCs were also found to activate glucose uptake and effectively reduce blood glucose levels in both type 1 and type 2 diabetic mouse models [29,30]. LysoPCs were defined as a novel ligand for G protein-coupled receptor 119 (GPR119). Oleoyl lysophosphatidylcholine (LPC 18:1) binds to the membrane receptor of GPR119 on β-cells, regulating insulin secretion through a protein kinase A (PKA)-related signaling pathway [31,32], and promotes glucose-stimulated insulin secretion in murine NIT-1 insulinoma cells [30,31]. The mechanism may be that LysoPCs with a covalently bonded molecule of p-anisic acid at the sn-1 position induces glucose-stimulated insulin secretion and intracellular calcium flux. Studies have shown that three G-protein-coupled receptors GPR40, GPR55, and GPR119, are targeted by LysoPCs [33]. LysoPCs are also involved in reducing fat deposition and food intake. In the present study, PIGinH11 pigs had decreased levels of lysoPCs and increased blood glucose levels, supporting the results of previous studies.

PSs are mainly distributed inside the bilayer membrane and have a net negative charge that contributes to the fluidity of the membrane. PSs are involved in the regulation of oxidative stress, apoptosis, and coagulation processes, and are associated with inflammation and autoimmune diseases including preeclampsia and systemic lupus erythematosus [34]. Various clinical plasma biomarkers have been reported in metabolomic and lipidomic studies related to metabolic disorders. However, few data related to PS have been reported. Chronic inflammation of adipose tissue is one of the typical features in the development of obesity and T2DM. Tulipani et al. [18] reported that extremely obese individuals with or without evidence of pre-diabetes had significantly elevated plasma PS (38:4). Grace et al. [35] also found that postprandial plasma PS (38:4) was significantly increased in patients with T2DM after physical activity. Recent research on Duroc pigs fed a high-calorie diet [36] found that PS (O-38:1), PS (P-39:0), PS (P-40:1), PS (O-40:5), PS (43:0), and PS (43:1) were significantly increased in pigs with prepubertal obesity, among which PS (21:0/22:0) was positively correlated with pig visceral fat content and human BMI. In this study, seven PSs were significantly changed between groups. The dietary intervention had different effects on plasma PS contents in PIGinH11 and WT pig groups, respectively. The PSs value was significantly decreased in WT after HFHSD, two of them reduced to almost 10% of baseline, the other five PS (O-20:0/16:0), PS (13:0/22:0), PS (21:0/22:1), PS (O-18:0/15:1) and PS (22:2/22:4) were lower than 1%. While the changes of these PSs range from 0.92 to 1.46 folds in PIGinH11 pigs for dietary intervention. Due to the value of these PSs in PIGinH11 pigs being 2-3 times that of WT pigs in the baseline, folds between groups increased to 10- to 100 after dietary intervention. The PSs activate macrophages through T-cell membrane protein 3 (TIM3), anoctamin 4 (ANO4), and TMEM16, promoting inflammation or autoimmune responses [34]. Our previous transcriptome study on PIGinH11 pigs suggested that metabolic inflammation in adipose tissues and the liver might be initiated by the activation of CD8+ T cells triggered by antigen presentation. This study supplemented that the TMEM16F-mediated PSs externalization induced by cellular injury may also play an important role in the development of inflammation by binding to macrophage-specific receptors.

Thirdly, plasma secondary bile acids were decreased in PIGinH11 pigs. In addition to intestinal lipids emulsification, bile acids are also involved in the homeostasis of blood glucose, triglycerides, and cholesterol. Previous studies have shown that hyocholic acid (hCA) has a protective effect against diabetes mellitus. In this study, seven species of bile acids differed between PIGinH11 and WT pigs. Among them, secondary bile acids including hCA, deoxycholic acid 3-glucuronide, glycodeoxycholic acid, ursodeoxycholic acid, and trihydroxy-5b-cholanoic acid were significantly lower in PIGinH11 after dietary intervention than that of WT group at baseline. The most abundant bile acid detected in plasma was hCA, its content in PIGinH11 pigs was 36.9% (*p* = 0.05) of WT pigs at baseline. hCA was proven to regulate blood glucose in pigs and diabetic mouse models [37]. hCA promotes GLP-1 secretion by simultaneously activating G protein-coupled bile acid receptor 1 (GPBAR1, also refers as TGR5) and inhibiting Farnesoid X receptor (FXR). Hydroxycholic acid also improves glucose homeostasis through TGR5 and FXR signaling mechanism [38,39]. Many studies found that bile acids serve as a hormone to control various biological processes such as energy metabolism, cholesterol/ bile acid metabolism, immune responses, and glucose/ lipid metabolism by binding to their endogenous receptors, including FXR and TGR5. If deficiency of bile acid receptors may induce metabolic syndromes such as obesity, insulin resistance, hyperglycemia, and hyperlipidemia. Numerous studies have consistently reported bile acid signaling pathways alteration in T2DM patients. Bile acids have been shown to activate TGR5 in intestinal L cells and then enhance the secretion of GLP-1 [40]. Studies show that TGR5 expression was significantly decreased in the distal ileum and ascending colon in DM RAT. FXR expressions in ascending colon were also decreased in DM RAT. Correlation analysis found correlations between plasma total bile acids TBA and GLP-1A or fibroblast growth factor 15 (FGF15). In the colon, GLP-1A was correlated with TGR5 mRNA expression and FGF15 was correlated with FXR mRNA expression [41]. Some finding reveals that pre-diabetes patients are associated with lower levels of hCA species in feces. Plasma hCA levels increase in the patients after gastric bypass surgery. hCA can predict the remission of diabetes two years after surgery. Two independent, prospective cohorts (n = 132 and n = 207) replicated these results, they found respectively that plasma hCA species are strong predictors for metabolic disorders in five and ten years. All these findings prove the association of hCA species with diabetes and demonstrate that using hCA profiles to assess the future risk of developing metabolic abnormalities is feasible [38].

## 5. Limitations of the Study

The limitation of this study is, firstly, the observation period is only two months, which is a short period of time; further delay in observation is needed to determine the elevated blood glucose stability, during which the occurrence of complications can be observed. Secondly, the development of metabolomic approaches has brought great progress to the screening of biomarkers for metabolic diseases, however, explaining the specific pathways linking the biomarkers and diseases remains a challenge [42,43]. Thirdly, a specific metabolomic characteristic may be affected by complex factors such as specific organs and tissues, dietary intake, or interaction between the microbiome and environment [44]. Since our study is based on observational data, the interpretation of the results does not necessarily represent causation. This problem can be solved by combining genetic, proteomic, and/or other levels of data, and designing point-to-point verification experiments.

## 6. Conclusions

The differential plasma metabolites between PIGinH11 and WT group from plasma metabolome and lipidome formed a network that contributed to the development of insulin resistance with three characteristics (Figure 4G). Firstly, various fatty acylcarnitines were significantly increased, indicating that fatty acid β-oxidation in PIGinH11 pigs might be enhanced, and the accumulation of by-products in the TCA cycle originating from fatty acids metabolism might interfere with catabolism of glucose, BCAAs, and AAAs. The accumulation of methyl donors such as Methionine and Betaine promoted carnitine production. Through the step-by-step amplified effect of methionine-carnitine-acylcarnitine metabolism, fatty acid β-oxidation in the mitochondria increased, thereby disturbing glucose and amino acid metabolism and exacerbating insulin resistance. Secondly, de novo synthesis of DGs from fatty acids was decreased, as a large proportion of fatty acids might be consumed by acylcarnitine synthesis. Meanwhile, PNPLA3^I148M^ inhibited the decomposition of polyunsaturated acyl TGs to DGs and limited the production of PCs and PEs, thus affecting the structure and function of cell membranes and leading to cell damage. The reduction of PCs limited sphingolipid synthesis, thereby leading to accumulations of ceramide and sphingosine, which play important roles in insulin resistance and inflammation. Finally, the reduction of bile acids and LysoPCs leads to the metabolic disturbance of glucose, amino acids, and lipids. The regulation of metabolic homeostasis, insulin sensitivity, and anti-inflammatory effects by these metabolites was undermined. Meanwhile, a group of PSs was also increased due to metabolic disturbances, which might promote the development of chronic inflammation by activating macrophages.

## Figures and Tables

**Table 1:** The body weight and plasma biochemical parameters of wild-type and PIGinH11 pigs at the baseline and after HFHSD feeding.

Var	WT-0	WT-2	TG-0	TG-2	d_WT_	d_TG_	*P*_WT-2/WT-0_	*P*_TG-2/TG-0_	*P*_TG-0/WT-0_	*P*_TG-2/WT-2_
Weight (kg)	29.1 ± 5.1	50.2 ± 6.7	29.5 ± 5.2	48.8 ± 7.5	21.2 ± 7.9	19.3 ± 5.9	<0.001	<0.001	0.852	0.662
Glu (mmol/L)	4.8 ± 0.7	6.1 ± 1.6	7.4 ± 1.7	7.6 ± 0.8	1.3 ± 1.4	0.1 ± 1.4	0.013	0.813	<0.001	0.022
Ins (μIU/mL)	40.4 ± 15.9	64.3 ± 13.0	101.6 ± 56.7	62.0 ± 10.6	23.9 ± 15.2	−39.6 ± 52.7	<0.001	0.041	0.008	0.662
Tg (mmol/L)	0.4 ± 0.1	0.4 ± 0.2	0.5 ± 0.1	0.4 ± 0.1	0.0 ± 0.2	−0.0 ± 0.1	0.872	0.216	0.166	0.651
Tc (mmol/L)	2.0 ± 0.5	2.6 ± 0.5	1.9 ± 0.5	2.4 ± 0.5	0.6 ± 0.6	0.5 ± 0.5	0.015	0.009	0.656	0.325
Hdl (mmol/L)	0.8 ± 0.2	1.4 ± 0.3	0.9 ± 0.1	1.1 ± 0.2	0.5 ± 0.3	0.2 ± 0.1	<0.001	<0.001	0.906	0.023
Ldl (mmol/L)	0.9 ± 0.2	0.7 ± 0.2	0.7 ± 0.3	0.7 ± 0.2	−0.1 ± 0.3	−0.1 ± 0.3	0.116	0.349	0.298	0.488
GLP-1 (pmol/l)	0.8 ± 0.2	0.6 ± 0.3	0.6 ± 0.3	0.9 ± 0.5	−0.2 ± 0.2	0.3 ± 0.6	0.008	0.130	0.025	0.113

Data were shown as mean ± SD. d_WT_, the difference value between WT-2 and WT-0. d_TG_, the difference value between TG-2 and TG-0. *P* _WT-2/WT-0_, the *p*-value for the comparison between WT-2 and WT-0, as well as *P*_TG-2/TG-0_, *P*_TG-0/WT-0_, *P*_TG-2/WT-2_. Glu, glucose; Ins, insulin; Tg, triglycerides; Tc, total cholesterol; Hdl, high-density lipoprotein cholesterol; Ldl, low-density lipoprotein cholesterol; GLP-1, glucagon-like peptide-1.

**Table 2:** The differential features detected with untargeted metabolome and lipidome between wild-type and PIGinH11 pigs at the baseline and after HFHSD feeding.

Group	Metabolomics			Lipidomics		
	All	Up	Down	All	Up	Down
WT-2 vs. WT-0	273	139	134	228	106	122
TG-2 vs. TG-0	269	120	149	244	144	100
TG-0 vs. WT-0	155	56	99	226	103	123
TG-2 vs. WT-2	172	62	110	222	127	95

## Data Availability

The data presented in this study are available in Tables S1–S6.

## Supplementary Materials

The following supporting information can be downloaded at: https://www.mdpi.com/article/10.3390/metabo12121164/s1, Table S1: Changed compounds meas_pos of untargeted metabolomics; Table S2: Changed compounds meas_neg of untargeted metabolomics; Table S3: Changed compounds meas_pos of untargeted lipidomics; Table S4: Changed compounds meas_neg of untargeted lipidomics; Table S5: Enrichment of differential metabolites between groups; Table S6: List of main differential metabolites between groups.
